# Synchronisation strategies in T2-weighted MR imaging for detection of liver lesions: Application on a nude mouse model

**DOI:** 10.2349/biij.3.4.e53

**Published:** 2007-10-01

**Authors:** L Baboi, L Milot, C Lartizien, C Roche, J-Y Scoazec, F Pilleul, O Beuf

**Affiliations:** 1 CREATIS-LRMN, CNRS UMR 5220, Inserm U630, INSA-Lyon, Université de Lyon, Université Lyon 1, Villeurbanne, France.; 2 Hospices Civils de Lyon, Département d’Imagerie Digestive, CHU Edouard Herriot, Lyon, France.; 3 Inserm U865, Faculté de médecine RTH Laennec, Lyon, France.

**Keywords:** mice liver, high-field MRI, synchronisation, motion artifacts, T2-weighted contrast image

## Abstract

**Aim:**

The objective of this work was to propose original synchronisation strategies based on T2-weighted sequence performed on a small animal MRI spectrometer in order to improve the image contrast and detect mouse liver lesions at high magnetic field.

**Materials and Methods:**

The experiments were performed *in vivo* at 7T using a 32 mm inner diameter cylindrical volumetric coil for both RF emission and reception. A sensitive pressure sensor was used to detect external movements due to both respiration and heart beats. The pressure sensor was interfaced with a commercial ECG Trigger Unit to use dedicated functionalities (trigger levels, delays and window). To enable T2-weighted imaging with minimised T1 effects, an acquisition strategy with controlled TR spanning over several respiratory cycles was developed. With this strategy, the slices were acquired over several respiratory periods.

**Results:**

The acquisition, performed over several respiratory periods, enables a longer TR than the typical mouse respiratory period. The image contrast is controllable and independent of the respiratory period. The heavily T2-weighted images obtained with the developed strategy allow better visualisation of lesions at high magnetic field. Moreover, double respiratory and cardiac synchronisation, based on a unique sensitive pressure sensor, improves image quality with less motion artifacts, especially in the ventral liver region. The total slice number is independent of respiratory period and thin slices can be acquired to cover the whole liver.

**Conclusion:**

The developed strategy enables high quality pure T2-weighted imaging with minimal motion artifacts. This strategy improves T2-weighted image contrast and quality, especially at high magnetic field, on animals with short respiratory periods. The strategy was demonstrated using a mouse model of liver lesions at 7T. This protocol could be used to carry out a longitudinal follow-up.

## INTRODUCTION

Magnetic resonance imaging is an established imaging method for the evaluation of liver diseases. In clinical practice, MR imaging examination of the liver uses a combination of breath-hold T1-weighted gradient images and T2-weighted images, including gadolinium enhancement with acquisition of multiple phases. In order to avoid blurring and ghost artifacts due to respiratory motions, several techniques for T2-weighted imaging have been proposed [1]. Breath-hold T2-weighted imaging is feasible with a long echo train to reduce breath-hold times. This technique is associated with a short inter-echo spacing to minimise artifacts due to T2-dependent signal decay occurring with a long echo train. The technique has been proven to be superior to a free-breathing conventional spin-echo (SE) sequences in the detection of hepatic tumours at 1.5 T [2, 3]. The benefits of breath-hold T2-weighted imaging include better lesion conspicuity and reduced blurring by an order of magnitude in image acquisition [2, 4]. An alternative to the breath-hold approach is the respiratory-triggered fat-suppressed (FS) fast SE imaging. This technique is more accurate than the free-breathing with SE sequence or breath-hold with fast SE sequences, with improved small lesion conspicuity [5, 6]. In small animal models, liver study on MR imaging requires specific adaptation including strong constraints in the conditioning of the animals and the spatial resolution [7]. It is mandatory to control the anaesthesia and the temperature by measuring the physiological signals. Conventional SE T2-weighted sequence has the potential to provide high image contrast and spatial resolution if motion artifacts are suppressed. Conventional procedure for small animals consisted of synchronisation of T2-weighted imaging with the respiratory cycle [8-11]. However, slices are acquired on each respiratory cycle, and the number of acquisition slices is limited by the short respiratory period T_resp_ (typically in the range of 0.5 s to 1.5 s), leading to insufficient liver coverage. Moreover, the image contrast is not freely controllable because TR is given by T_resp_ and is usually unsuitable for T2-weighted image contrast, especially at high magnetic field [12].

In this study, we performed a prospective qualitative comparison between conventional acquisition T2-weighted imaging and three increased evolutions of T2-weighted images to improve detection of liver lesions and to determine which acquisition strategy is best for repeated examinations during a longitudinal study.

## MATERIALS AND METHODS

### Animal model

Female athymic nu/nu CD-1 nude mice between 7 and 11 weeks old, obtained from Charles River Laboratories (L'Arbresle, France), were used. The animals were bred and maintained in a filtered environment. Cages, food and bedding were sterilised in the autoclave. The experimental protocol was approved by the Animal Care and Use Committees of the University Claude Bernard Lyon 1. The intestinal STC-1 cell line, a gift from D. Hanahan through the courtesy of A. Leiter (New England Medical Center, Boston, MA), was derived from an endocrine tumour developed in the small intestine of a double transgenic mouse obtained by crossing two lineages expressing the rat insulin promoter linked, respectively, to the simian virus 40 large-T antigen and to the polyomavirus small-t antigen [13]. STC-1 cells were maintained in DMEM supplemented with 5% foetal calf serum (FCS), 2 mmol.L^-1^ glutamine and antibiotics (100 UI.mL^-1^ penicillin plus 50 mmol.L^-1^ streptomycin). Animals were anaesthetised with pentobarbital prior to all surgical procedures. A 50 µl solution, containing STC-1 cells adjusted to a final concentration of 5.10^7^ cells.mL^-1^, was injected into the spleen of adult female mice after abdominal sterilisation with iodine and alcohol swabs [14].

### Anaesthesia

The anaesthesia protocol was conducted with an approved system (Minerve, Esternay, France). First, mice were placed in an anaesthesia induction box with 2 - 2.5 % isoflurane gas (Laboratoire Belamont, Boulogne Billancourt, France) administered at 1 L/min rate. Then the animals were placed in a supine position on a dedicated plastic bed [15]. To maintain the anaesthesia during the acquisition, the mouse’s nose was introduced into a face cone mask delivering anaesthetic gases (1 - 2 % isoflurane with a mixture of 70% oxygen and 30% air at 0.6 to 1 L/min).

### Pressure sensor and triggering device

The cardiac and respiratory movements were detected using a home-made air pillow placed next to the thorax and upper abdomen. The air pillow was connected with a plastic tube to a sensitive pressure sensor (reference DCXL01DN, Honeywell, Freeport, IL, USA) and detected movement due to respiration and heart beats. The pressure range of this ultra low pressure sensor was within 1 inch height of H_2_O. Pressure sensor was interfaced (connection and power supply) with an ECG Trigger Unit HSB-T (Rapid Biomedical, Würzburg, Germany), in order to use the adapted functionalities of this trigger unit (amplification, filtering, trigger level, trigger delay and trigger window). Because the trigger unit used had no screen display, signals were displayed using a digital oscilloscope (Tektronix TDS 2014, Beaverton, OR, USA).

**Figure 1:**
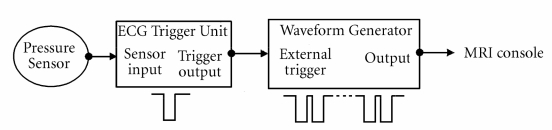
Schematic of connection for sensor and devices used for triggering. An air pillow was connected to a pressure sensor for both respiration and heart motion detection. The pressure sensor was interfaced with the ECG Trigger Unit HSB-T. This device ensures the amplification and filtering of input signal as well as the adjustment of the trigger level, trigger delay and trigger window. The trigger pulse generated by the ECG unit is used by the waveform generator to send a burst of trigger pulses to the MRI console.

To generate multiple trigger pulses to the MR console using a single output trigger pulse from the ECG Trigger Unit, or to control the minimum delay between two trigger pulses, we used a waveform generator 33220A (Agilent Technologies, Palo Alto, CA, USA). A burst of one or more square pulses was generated with adapted number of cycles, depending on the respiratory period. The connection scheme between devices is shown in Figure 1.

### Imaging protocol

The experiments were performed *in vivo* at 7 T on a Biospec system (Bruker, Ettlingen, Germany) equipped with a shielded gradient system (400 mT/m gradient strength and 120 mm diameter). For both RF emission and reception, a cylindrical volumetric coil with 32 mm internal diameter (Rapid Biomedical, Würzburg, Germany) was used. A FS multiple SE sequence with three echoes (TE=20, 40, 60 ms) was used with the following parameters: 30 x 30 mm^2^ FOV, 256 x 256 matrix, 0.5 mm slice thickness and 17 kHz receiver bandwidth. Images were acquired in the axial plane, using two axial saturation bands placed on both sides of the acquisition box to reduce flow artifacts. The minimum delay between two consecutive slices with these sequence parameters was 90 ms. The delay between two consecutive slices was defined as the inter-slice time (T_is_).

### Triggering strategies

#### Method I: Conventional triggering strategy

**Figure 2:**
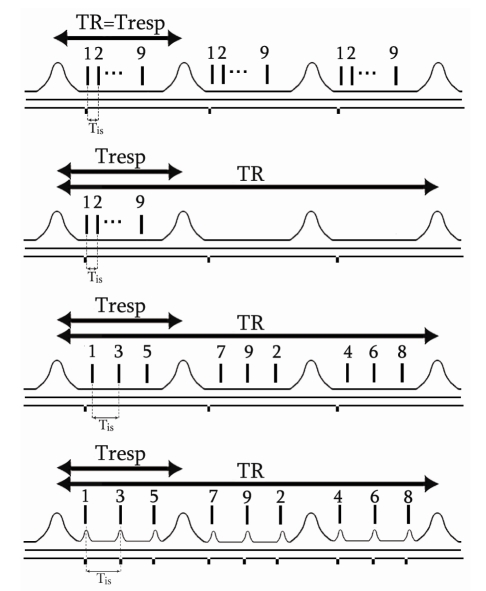
Diagrams of different synchronisation and slice acquisition strategies used to obtain T2-weighted contrast images. The trigger pulses sent by the trigger unit to the MRI console as well as the excitation timing of each slice is indicated and illustrated for 9 slices: (a) Conventional triggering strategy for T2-weighted contrast images. The repetition time is equal to the respiratory period with TR = T_resp_. All slices are acquired within one respiratory cycle (non interleaved case); (b) Heavier T2-weighted image contrast is obtained with minimum controlled TR imposed between two slice packages; (c) New acquisition strategy with balanced slice acquisitions. A trigger pulse is generated for every single slice and acquisition of slices is spanned over several respiratory cycles; (d) Dual cardiac and respiratory triggering with balanced acquisition over several respiratory cycles. The time between two consecutive slices is called inter-slice time (T_is_).

The conventional triggering procedure consists of synchronising all slices, starting from one single trigger pulse. The trigger time point is adjusted manually for each mouse by setting the trigger level and delay on respiration signal to occur between the end of the expiration and the beginning of inspiration. Using this basic technique, all slices should be acquired within the expiration period from one cycle. TR is determined by the respiratory period with TR = T_resp_. For every trigger pulse, a single line of k-space is acquired for all slices [16]. The trigger signal and the acquisition period over the respiratory waveforms are shown in Figure 2a.

#### Method II: Uncoupling TR from T_resp_

To perform heavily T2-weighted contrast images with minimal T1 effects, TR has to be increased to be longer than the respiratory period. This was carried out by decoupling the respiratory period T_resp_ from the repetition time TR by introducing a minimum delay between two trigger pulses (Figure b). As in the previous method, all the slices are acquired within one respiratory cycle but not withevery single cycle. With a minimum delay between two trigger pulses of 3 s, signal acquisition is performed every two to six respiratory cycles depending on mouse T_resp_ value.

#### Method III: Balanced acquisitions over several respiratory periods

Similar TR imposed with previous evolution can be obtained by spanning multiple slice acquisitions over several respiratory cycles (N_resp_). While TR = N_resp_ x T_resp_, the effective TR is almost independent of T_resp_ and relatively constant between animals by choosing an adapted number of respiratory cycles. For this method, an independent trigger pulse is generated for every single slice and all the slices are acquired within several respiratory cycles corresponding to desired repetition time TR.

At the end of every expiration cycle, a burst of trigger pulses is generated using the waveform generator. The burst of trigger pulses consists of a series of square pulses with 105 ms delay between each pulse sent to the MRI console. The ‘low’ level of the square pulses is in the range of zero to 0.8 volts, and the ‘high’ level is in the range of 2 to 5 volts according to TTL (transistor-transistor logic) requirements for trigger console input. The delay between two pulses was set slightly longer than the minimum delay needed to acquire one slice (90 ms). The number of square pulses generated within one burst was chosen based on the respiratory period in order to fully use the expiration period. For each trigger pulse, a unique slice with a single k-space line was acquired. Figure 2c is a schematic diagram showing the principle of this triggering and acquisition strategy over the respiratory waveforms. For instance, in a sequence with 9 slices, the slices can be divided into 3 groups. Each slice group is acquired within one respiratory cycle: the slices ordered (interleave case) and numbered as 1, 3, 5 are acquired in the first cycle, the slices numbered 7, 9, 2 in the second cycle and the slices numbered 4, 6, 8 in the third cycle. For a mouse with a respiratory period of 1.5 s, 36 slices can be successfully acquired by exciting 12 slices (with a burst of 12 trigger pulses) per respiratory cycle, which means that 3 respiratory cycles are needed to cover the entire liver, leading to an effective TR of 4.5 s. This synchronisation and acquisition scheme allows an increasing total number of slices, by increasing the effective TR, depending on what is required. Thus in our specific case, 36 slices were acquired to cover the whole mouse liver with thin slices of 0.5 mm thickness.

#### Method IV: Dual cardiac-respiratory triggering with balanced acquisitions

**Figure 3:**
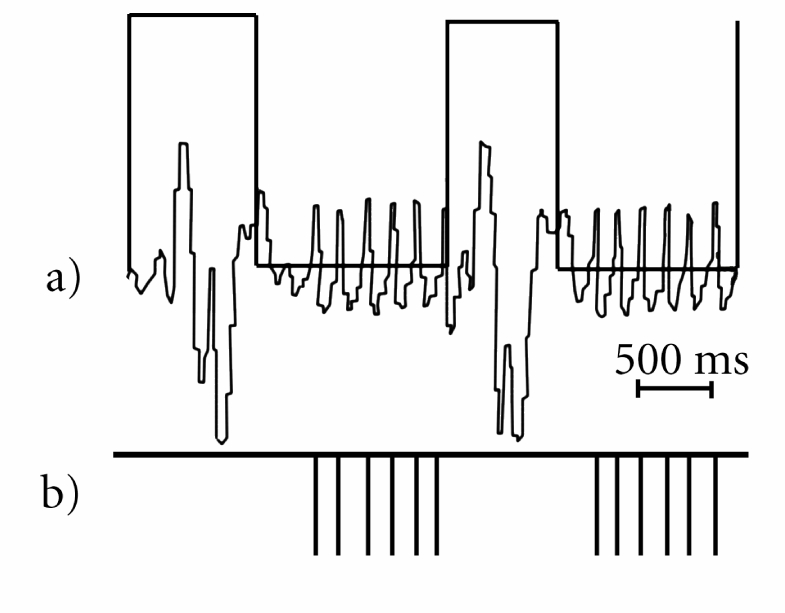
Example of the setup for dual cardiac-respiratory triggering: (a) Signal from the pressure sensor with acquisition window enabled between two respiratory cycles; (b) Trigger pulses sent to MRI console based on cardiac cycle.

Due to the close proximity of the heart, heartbeats can generate motion artifacts in the upper part of the liver. To avoid liver contamination by heart motion artifacts, a dual cardiac-respiratory triggering was used. In this strategy, the variability of the respiratory rate was determined prior to MR acquisition and the scanning period was chosen to be shorter than the shortest respiratory period. Thus, a safe number of cardiac cycles that were likely to occur for all respiratory cycles during MR acquisition was used. The synchronisation and acquisition scheme is shown in Figure 2d. An example of real signals and setup (trigger level, acquisition window) for dual cardiac-respiratory triggering is shown in Figure 3.

### Qualitative and quantitative evaluation

Image quality obtained with the four different triggering methods was assessed, taking into account the presence of artifacts and overall image quality as well as the contrast between smaller hepatic lesions and liver tissue. Additionally, the signal-to-noise ratio (SNR) was measured in hepatic parenchyma and in large (> 1 mm in diameter) lesions. The contrast-to-noise ratio (CNR) between lesions and parenchyma was also calculated. Both parameters were assessed and corrected to the square root of the total scan time for comparisons between the conventional triggering technique and the dual cardiac-respiratory triggering with balanced acquisitions.

## RESULTS

**Figure 4:**
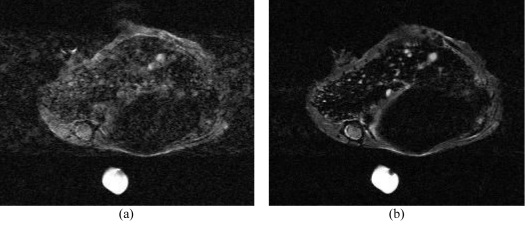
Images of mice liver obtained at D21: (a) without any synchronisation with TR ≈ 1.5 s; (b) with respiratory triggering and slice excitation spanned over three respiratory cycles (TR ≈ 3 × T_resp_ ≈ 4.5 s).

**Figure 5:**
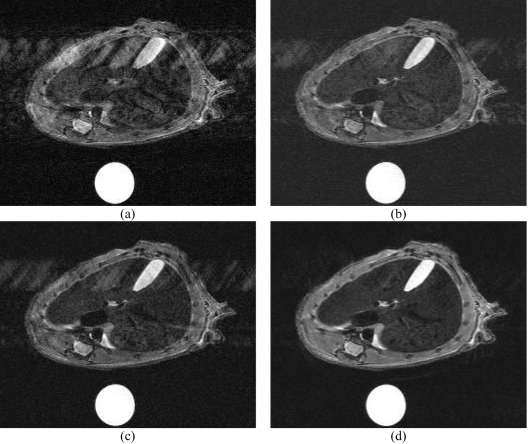
Images of mice liver (T_resp_ = 1.5 s; 12 slices; slice thickness 1.5 mm) obtained on normal specimen: (a) without synchronisation (TR = 1.5 s; TA = 5 min 20 s); (b) with conventional respiratory strategy and a long controlled TR (TR ≈ 3 s; T_is_ = 90 ms; TA = 9 min); (c) with respiratory triggering strategy with balanced acquisitions over several respiratory periods (TR ≈ 3 × T_resp_ = 4.5 s; T_is_ = 105 ms; TA = 9 min); (d) with dual cardiac and respiratory triggering with balanced slice acquisitions (TR = 4.5 s; T_is_ ≈ 200 ms; TA = 17 min).

The analysis of the nude mice hepatic lesion images obtained from the various synchronisation schemes enabled us to qualitatively characterise each proposed strategy. Efficient respiratory synchronisation is mandatory to obtain reasonable image quality that allows the detection of small lesions. An illustration of image degradation due to motion artifacts with and without synchronisation on respiratory motion is shown in Figure 4. Representative images of the inferior epigastric area, acquired with three out of four investigated strategies, are shown in Figure 5 on normal specimen. On T2-weighted images, the gall bladder appears in hyperintensity signal. In the presence of motion, this high intensity signal will propagate along the phase encoding direction. Without respiratory triggering, the motion artifacts are present (Figure 5a). In the conventional respiratory strategy with a long controlled effective TR, a good T2-weighted contrast image is achieved, but with motion artifacts at the gall bladder due to cardiac movements (Figure 5b). All the slices (limited to about 12 slices) are acquired within one respiratory cycle but not with every single respiratory cycle. This strategy is clearly not efficient since scanning is performed only during the expiration delay of one cycle over three. Using the respiratory triggering strategy with balanced acquisitions over several respiratory periods, the artifacts and the image contrast are equivalent to the previous strategy but it has the advantage that the number of acquirable slices increases three fold (up to 36 slices). The additional cardiac synchronisation significantly reduces the remaining motion artifacts at the cost of an increase in scan time.

**Figure 6:**
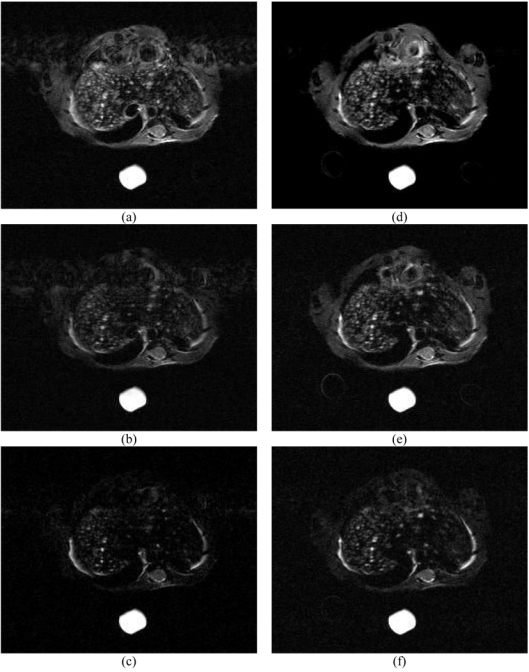
Multi-echo images (T_resp_ ≈ 2 s; 36 slices; 0.5 mm slice thickness) and slice excitation spanned over several respiratory cycles obtained at D21: (a, c, e) with respiratory triggering (TR ≈ 3 × T_resp_; T_is_ = 105 ms; TA = 21 min) for TE = 20, 40, 60 ms; (b, d, f) with cardiac and respiratory triggering (TR ≈ 6 × T_resp_; T_is_ = 155 ms; TA = 35 min) for TE = 20, 40, 60 ms.

For the detection and the characterisation of liver lesions, multi-echo imaging with dual cardiac and respiratory triggering is very useful, especially in the ventral liver region where motion artifacts are generated by the heartbeat (Figure 6).

With an average T_resp_ of 1.5 s and a variable effective TR depending on T_is_, the total acquisition time was in the range of 20 to 38 min. The main parameters and characteristics of different strategies evaluated in this work are summarised in Table 1.

**Table 1:** Summary of different synchronisation strategies with associated parameters of interest given for an average respiratory period T_resp_ ≍ 1.5 s

**Synchronisation methods**	**Typical number of slices within one scan **	**Effective TR**	**Inter-slice time T_is_ (ms)**	**T2-Weighted contrast **	**Motion artifact type**	**Average scan time (min)**
Conventional respiratory triggering strategy	10 to 12	T_resp_	T_is_ = 90 ms	-	Cardiac	6-7
Conventional respiratory strategy with long controlled TR	10 to 12	≍ 3 x T_resp_	T_is_ = 90 ms	+	Cardiac	20
Respiratory triggering strategy with balanced acquisitions over several respiratory periods	30 to 36	≍ 3 x T_resp_	T_is_ = 105 ms	+	Cardiac	20
Dual cardiac and respiratory triggering with balanced slice acquisitions	30 to 36	≍ 6 x T_resp_	T_is_ ≍ 200 ms	++	Free	38

The ratio of the SNR corrected for acquisition time using the dual cardiac-respiratory triggering to balanced acquisitions with the conventional triggering technique was constant with a mean value of 1.01 ± 0.02. However, CNR corrected for acquisition time using the dual cardiac-respiratory triggering with balanced acquisitions increased by 41 ± 11%, 12 ± 2%, and 15 ± 4% for 20, 40 and 60 ms TE respectively, compared to the conventional triggering technique.

## DISCUSSION

An MRI protocol adapted to the study of liver tumours of nude mice, including ‘strong’ constraints on the conditioning of the animals, was established. In a magnetic environment and with restricted space, we administered anaesthesia, controlled the animal body temperature and measured the cardiac and respiratory signals. Conventional triggering and different synchronisation strategies not available in commercial high field small animal MRI spectrometers were assessed.

The regular protocol consists of T2-weighted imaging synchronised with the respiratory motion. In order to significantly reduce motion artifacts, images within the late expiration period were acquired. This conventional method has several limitations: limited number of slices (the number of slices that can be acquired is restricted in between the trigger pulse and the next inspiration event), significant cross-talking due to the time reduction between two slices, and poor T2-weighted contrast. The limited number of slices allowed to cover the liver volume can be circumvented with larger slice thickness but this leads to partial volume effects. Finally, the choice of the image contrast is limited by the fact that TR is controlled by T_resp_. Thus, image contrast is not freely controllable and TR is unsuitable for the required T2-weighted image contrast [17-19] especially at high magnetic field (4.7 T and more) due to the longitudinal relaxation time T1 increase with magnetic field.

With the second method, suitable T2-weighted image contrast was obtained by uncoupling TR and T_resp_. Despite the improved contrast, the slice number was still limited and not optimal in terms of effectiveness, since scanning is performed only during the expiration delay of one cycle over three (Figure 2b).

With the third method, the number of slices was increased due to the fact that the slices were divided into a few groups. Thus the entire liver was covered with thinner slices, resulting in reduced partial volume effect. Another advantage of this slice repartition is the reduction of the possible cross-talking due to the increased time separation between two adjacent slices. Furthermore, the decoupling between T_resp_ and the effective TR is preserved and thus the T2-weighted contrast is almost as freely controllable as with the second method.

The dual cardiac-respiratory triggering strategy with balanced acquisitions leads to equivalent T2-weighted image contrast and quality far from the heart but significantly improves image quality and detection of the hepatic lesions in the liver dome region at close proximity to the heart. To our knowledge, no papers have reported improved T2-weighted contrast images with dual respiratory and cardiac synchronisation performed on a small animal MRI system at high magnetic field.

The various synchronisation techniques described in this paper do not depend on the imaging sequence used and could be applied to other sequences such as RARE (Rapid Acquisition Relaxation Enhancement) for T2-weighted contrast imaging.

On most commercial synchronisation units, heart synchronisation is usually performed based on Electrocardiogram (ECG) signals [7, 20]. During a scan, RF pulses and gradient switching induce eddy currents disturbing the ECG signal [21]. For small flip angles, the ECG signal can be easily filtered to recover a usable signal for triggering and to perform, for example, FLASH acquisitions in a CINE mode. ECG filtering is much more challenging using SE or RARE sequence with additional FS pulse or saturation bands. The pressure sensor used for respiratory and cardiac triggering was deported outside the RF coil, the gradient coil and the magnet bore. Pressure signal is uncorrupted by the eddy currents induced by RF pulses or gradient switching and thus it can be used with EPI sequence and large flip angles with good reproducibility for *in vivo *experiments.

At this time, the acquisition speed for dual cardiac-respiratory triggering with balanced acquisitions is limited by T_is_ that is given by the cardiac cycle period in the 200 ms range. In order to increase acquisition speed without deteriorating image contrast and quality, we are currently modifying a sequence in order to excite two slices within one single heartbeat. The first slice located close to the heart will be then synchronised with it and the second one located far from the heart will be selected about 100 ms after the first one. With this ultimate evolution, we anticipate an image quality similar to full dual triggering with a drastic reduction of the scan time.

Finally, another way to increase acquisition speed would be to use parallel imaging techniques, using array coil with multiple elements.

## CONCLUSION

An original acquisition strategy for T2-weighted MR imaging of small animals at high magnetic field was developed. The method proposed allows acquisition of heavily T2-weighted liver images with respiratory and cardiac synchronisation. Dual cardiac and respiratory synchronization, using a unique sensitive pressure sensor, improved the image quality and detection of hepatic lesions especially in the liver dome region. The contrast was easily controllable due to the relative independence of the effective TR with the respiratory period. Moreover, this strategy allowed an important increase in the number of slices required for full liver coverage. The protocol will be used to carry out a longitudinal follow-up of hepatic lesions and to characterise the nude mouse model used before therapeutic follow-up.

## ACKNOWLEDGMENT

This work was supported by the Programme “Imagerie du Petit Animal CNRS-CEA 2005”. The authors would like to thank Lee Shoo Ming for the English language edition. The experiments were performed by authors on the Animage platform.
